# Feeling with a robot—the role of anthropomorphism by design and the tendency to anthropomorphize in human-robot interaction

**DOI:** 10.3389/frobt.2023.1149601

**Published:** 2023-06-02

**Authors:** Sarah Schömbs, Jacobe Klein, Eileen Roesler

**Affiliations:** Technische Universität Berlin, Berlin, Germany

**Keywords:** human-robot interaction, anthropomorphism, social robot, industrial robot, empathy, linguistic framing

## Abstract

The implementation of anthropomorphic features in regard to appearance and framing is widely supposed to increase empathy towards robots. However, recent research used mainly tasks that are rather atypical for daily human-robot interactions like sacrificing or destroying robots. The scope of the current study was to investigate the influence of anthropomorphism by design on empathy and empathic behavior in a more realistic, collaborative scenario. In this online experiment, participants collaborated either with an anthropomorphic or a technical looking robot and received either an anthropomorphic or a technical description of the respective robot. After the task completion, we investigated situational empathy by displaying a choice-scenario in which participants needed to decide whether they wanted to act empathically towards the robot (sign a petition or a guestbook for the robot) or non empathically (leave the experiment). Subsequently, the perception of and empathy towards the robot was assessed. The results revealed no significant influence of anthropomorphism on empathy and participants’ empathic behavior. However, an exploratory follow-up analysis indicates that the individual tendency to anthropomorphize might be crucial for empathy. This result strongly supports the importance to consider individual difference in human-robot interaction. Based on the exploratory analysis, we propose six items to be further investigated as empathy questionnaire in HRI.

## 1 Introduction

“We named ours Elly, our TALON. Yeah. And I talked to her, when I’m at the controls (...) I’d be coaxing her, “C’mon honey.” (laughs). They’re kind of part of the family, almost, you know?” tells Brady, an Explosive Ordnance Disposal (EOD) personnel and study participant of Carpenter (2013), p. 107, about his robot. A robot that is specially designed for unexploded ordnance tasks. Whereas this interview excerpt may seem odd at first glance, it gives a hint in what way humans perceive robots differently in comparison to other non-living objects (Duffy, 2003; Darling, 2016). And Brady’s impressions are no isolated incident. Several studies indicate that humans name their robots, refer to them as she or he, and actually *do feel* with their beloved companion (Sung et al., 2007; Sauppé and Mutlu, 2015). A phenomenon, commonly understood as anthropomorphization (Waytz et al., 2010). But whereas trusting a robot can be essential in various high-risk situations such as combat missions or a routine inspection (Groom and Nass, 2007; Hancock et al., 2011), empathy could be dangerous in these human-robot interaction (HRI) scenarios. For instance, anecdotal evidence suggests that the more soldiers might empathize with their robot, the greater influence it might have on their decision-making. Leading to scenarios in which soldiers could try to save their robots, while putting themselves in danger (Carpenter, 2013). The use case of military operations reveals the question of the extent to which a robot is seen and should be seen as a tool or a team member and to what extent this perception could affect empathic responses. After all, a tool is *something*, that helps you accomplish a task whereas a teammate is *someone*, probably worth saving. The EOD example has been used extensively because of its illustrative nature. However, a similar observation can be made with social robots entering various social environments. Sung et al. (2007) reported that people experienced grief when their beloved vacuum robot needed to be repaired. One participant narrated: “I can’t imagine not having him any longer. He’s my BABY!!” (Sung et al., 2007, p. 153). The authors collected written discourse from an online forum and interviewed current vacuum robot owners. During the study, participants explained that they worried and felt sorry for the robot when it got stuck underneath a chair or trapped someplace. Concerns, commonly understood as empathy.

But empathy is hard to grasp in HRI. Since situational empathy is an emotional response, a relatively large literature in HRI has investigated scenarios in which robots were being choked, punched or killed in order to provoke empathy (Riek et al., 2009; Rosenthal-von der Pütten et al., 2013a; Darling et al., 2015; Seo et al., 2015). Unfortunately, scenarios based on violence do not represent realistic HRI scenarios. They lack transferability, particularly in regard to social robots which are supposed to take on roles such as companions or tutors. Other studies assessed scenarios in which the robot feared memory loss (Seo et al., 2015), had to be put away in a box (Westlund et al., 2016) or needed participants’ donations (Onnasch and Roesler, 2019). In addition, it remains unclear which features actually do engender an empathic response. So far, the common ground seems to be that anthropomorphism - overall - enhances the chance of empathy. According to the hypothesis: a robot has to be as human as possible in order to induce human responses. However, both the vaccum robot and the military robot used in the study by Carpenter (2013) do not have an anthropomorphic appearance. Instead, both robots appear rather technical, if not mechanical. With this in mind, it is essential to understand which features evoke empathic responses towards even low anthropomorphically designed robots. This becomes particularly important in regard to the fact that direct collaboration is becoming more and more prominent not only in social but also in industrial settings, which are typically equipped with lower anthropomorphically designed robots (Millo et al., 2021). As the paradigm shift from cages to collaboration can be observed in all domains of HRI, empathy becomes an important domain-overlapping issue.

But what does this mean for social as well as industrial human-robot interaction? How can we induce empathy via design features if necessary, and avoid it if it might cause harm? To tackle these questions, we investigated the perception of and empathic responses towards robots, depending on the robot’s appearance (anthropomorphic vs. technical) and linguistic framing (anthropomorphic vs. technical). Since our study took place during a world-wide pandemic, the study was conducted online. But whereas past online studies often addressed simulated agents (Kwak et al., 2013; Seo et al., 2015), we used video-clips of the robot inspired by Rosenthal-von der Pütten et al., 2013a, filming real robot movements. Therefore, participants knew that they were interacting with a real-world existing robot and not a virtual character. To move away from the existing violent-paradigm as proposed by Onnasch and Roesler (2019), we used a task in which participants had to execute instructions given by the robot in order to accomplish the task successfully. Hence, the robot and participant were interdependent. Due to the pandemic and our online study design, psychophysiological measures could not be realized. Nevertheless, we developed an empathic choice scenario and modified the German version of the Toronto Empathy Questionnaire (TEQ-D) (Schönefeld et al., 2018). Thus, we were able to assess situational empathy with both subjective as well as more objective measures to investigate participants’ empathic responses.

## 2 Theoretical background

### 2.1 Empathy in human-human interaction

Empathy is commonly understood as the ability to put oneself in someone else’s shoes. It is the ability to not just experience what others might feel, but to understand these feelings without confusing them with one’s own (Decety and Lamm, 2006). Empathy is oftentimes associated with people being warmhearted, welcoming, kind and helpful. Character traits that one wishes for in a good friend, a good caregiver or a good companion. On the other hand, if someone lacks empathic concern, this person is most likely going to be perceived as cold, rational and egocentric. Someone you would rather not have as tutor or colleague. As can be seen, empathy is a disposition that is stable in a person. This is why it is often referred to as trait empathy and can be measured by using and evaluating various questionnaires (Davis, 1980). Besides trait empathy, there is situational empathy. Situational empathy is described as an immediate empathic response in a specific situation (Zhou et al., 2021). It can be measured by exposing a person to a specific situation and asking them about their experiences immediately after. Situational empathy can also be examined by studying empathic cues like facial expression, gaze or language (Kumano et al., 2011; Zhou et al., 2021). In addition, numerous psychophysiological methods such as heart rate or skin conductance can be indicative for situational empathic responses (Eisenberg et al., 1994; Holmgren et al., 1998). Cuff et al. (2016) reviewed past definitions of empathy and highlighted their inconsistency. Besides dispositional and situational empathy, the authors discussed several aspects that were raised in previous research and convincingly conclude that empathy consists of both - cognitive and affective elements. To their mind, cognitive empathy is the ability to understand what others feel, whereas affective empathy is based on the experience of emotions due to emotional stimuli. In addition, empathy does not have to involve a person to be present. According to Cuff et al. (2016), empathy can also be evoked retrospectly or by a third person, telling a story about a person who is not present. Furthermore, empathy can be additionally experienced towards artificial agents or fictional characters, such as robots.

### 2.2 Empathy in human-robot interaction

In HRI, research on empathy can be divided into two main areas: The first area deals with simulating empathy. In this case, empathy is a design feature and can be perceived as a robot’s trait. The robot simulates empathy and seems to be able to *feel* empathic concern for other beings (Alves-Oliveira et al., 2019). For instance, a robot could be capable of reading facial expressions and mirror them in order to be perceived as empathic (Hegel et al., 2006). The second area investigates how empathy towards robots can be triggered *in a person*. In this case, humans feel *with the robot* and try to put themselves in the robot’s shoes. One important trigger for empathy is anthropomorphism by design, however, the basis for the effectiveness of this approach is the individual tendency to anthropomorphize (Waytz et al., 2010).

#### 2.2.1 Anthropomorphization and anthropomorphism

To understand the phenomenon of anthropomorphization and anthropomorphism and their relationship, it is necessary to take a closer look at their definitions. The tendency to anthropomorphize is a human disposition that can be more or less pronounced. Waytz et al. (2010) describe the tendency to anthropomorphize as “stable individual differences in the tendency to attribute human-like attributes to nonhuman agents” (Waytz et al., 2010, p. 219). Furthermore, these stable differences can “map onto important judgements, decisions, or behaviors”. As mentioned beforehand, people tend to name their vacuums, attribute intentional behavior to them and even feel sorry for their beloved companion when *he* is stuck under the couch (Sung et al., 2007). Nonetheless, not all objects are anthropomorphized in the same way and not every human makes anthropomorphic attributions to the same degree. The tendency to anthropomorphize can differ in regard to the object it refers to (Chin et al., 2004; Złotowski et al., 2015). Anthropomorphism as a design implementation, however, describes the transfer of human-like features onto robots to, for instance, promote an intuitive and socially situated HRI and to increase acceptance (Duffy, 2003). Anthropomorphism can be implemented by various morphological aspects, two of which are linguistic framing and appearance (Onnasch and Roesler, 2021). Whereas linguistic framing appeals top-down to the mental model of the human that is interacting with the robot (Kopp et al., 2022a), the robot’s appearance triggers an anthropomorphization due to an alteration of the robot’s external features (e.g., human-like shaped body).

For instance, a robot can be named *Paul* and hence activate social, anthropomorphic frames or a robot can be presented as *the robot*, highlighting its tool-like nature (Kopp et al., 2022b). Moreover, a robot embedded in an anthropomorphic storytelling can be perceived as more likeable, intelligent and human-like (Rosenthal-von der Pütten et al., 2017). Similarly, a robot described as a helper instead of a competitor can be perceived as more sociable (Horstmann and Krämer, 2020). On the other hand, a robot can differ in its appearance. An anthropomorphic appearance describes a design that resembles humans or involves human-like features. An early study by DiSalvo et al. (2002) indicates that specific facial features such as eyelids increase people’s perception of humanness when interacting with robotic heads. As can be seen, a robot does not have to have a strong resemblance in order to be anthropomorphized. Oftentimes, an anthropomorphic appearance can be engendered by the simple use of a human’s shape or specific features (Phillips et al., 2018).

#### 2.2.2 Empathic responses to anthropomorphism

Both morphology aspects-linguistic framing and appearance-can be applied to change someone’s attitude towards a robot and how a robot is perceived (Roesler et al., 2021). Darling et al. (2015) explored the impact of linguistic framing on empathic behavior, using personified stories and stories of experience. To operationalize empathy, the authors investigated participants’ willingness to strike a previously framed robotic toy with a mallet. Here, time of hesitation served as a measurement to assess situational empathy. The results suggest that anthropomorphic framing could be used to increase emotional responses toward robots. Moreover, Nijssen et al. (2019) investigated whether participants feel like a robot deserves to be saved in a moral dilemma when being framed anthropomorphically. In addition to framing, Nijssen et al. (2019) manipulated the agent’s appearance on three levels: human, human-like robot, and machine-like robot. The moral dilemma involved situations in which a group of people is in danger of dying, but could be saved if the participant actively sacrifices the agent. If the participant decides to not perform the action, thus not sacrifice the agent, the agent remains unharmed, whereas the group dies. The results revealed that participants were less willing to sacrifice the agent and more inclined to safe the agent when the agent had been described in a human-like way, regardless of the agent’s appearance. These findings indicate that linguistic framing could nudge people to feel sorry for robots.

Nevertheless, previous findings on linguistic framing cannot be generalized. Westlund et al. (2016) conducted an experiment in which a robot was being framed either anthropomorphically or machine-like. The goal of the experiment was to investigate how children act towards and perceive differently framed robots during a collaborative game. In the anthropomorphic condition, the robot was introduced as a friend. In addition, inclusive language was used like *you two* and *your friend*. In the functional condition, the robot was described in a tool-like manner, introduced as *robot* and referred to as *it*. During the game, the interaction was interrupted and the children were asked to put the robot away. Followed by the question whether the robot should be allowed to finish the game or not. Westlund et al. (2016) hypothesized that children would show significantly more social cues when the robot was being framed like a friend than a tool. Furthermore, the authors suggested that children would like the robot to finish the game. Nevertheless, results did not confirm such hypotheses. However, it is worth mentioning that several children were explaining their agreement to put the robot away by attributing social agency to the robot. The robot was described as tired and the box as its home. The latter illustrates the importance of investigating qualitative data in linguistically framed human-robot interactions. Moreover, the results support the assumption that framing could have a more subtle effect on a human’s perception of the robot. Post-hoc, the authors highlighted authority as an influencing factor: several children explained that adults were allowed to put a robot away. Onnasch and Roesler (2019) investigated participants’ willingness to donate money for an either anthropomorphically or technically framed robot. In line with Westlund et al. (2016), the results revealed contradicting insights. The authors hypothesized that an anthropomorphic framing would lead to a higher tendency to donate money to the robot. Unexpectedly, the results revealed that participants, who received a technical framing, donated more money to the robot and hence displayed more pro-social behaviour. To investigate these surprising findings, Onnasch and Roesler (2019) conducted a second experiment in which the functional value of the repair was made explicit. The authors assumed that an anthropomorphic framing could have disguised the functional need within the anthropomorphic condition and therefore the need to donate money. Indeed, results showed a slightly reversion of the effect. Still, both studies suggest that an anthropomorphic framing had no positive influence on participants’ pro-social behaviour an their willingness to donate money. Rather, both studies raise the question of whether framing has any effect at all when interacting with a humanoid robot (Onnasch and Roesler, 2019).

Focusing on a robot’s appearance, Riek et al. (2009) examined how people felt about robots being exposed to mistreatment by humans. During the experiment, participants watched 30-s film clips with robots from a different anthropomorphic spectrum varying from a vacuum robot to Alice, an adult-sized android. Within these film clips, robots were exposed to different kinds of harassment such as shouting or pushing. After the experiment, Riek et al. (2009) addressed empathy by asking the participants how sorry they felt for the robot followed by which of the robots they would save in an earthquake scenario. Results support the hypothesis that people feel more empathic with anthropomorphic looking robots than machine-looking ones. Seo et al. (2015) investigated how people felt with a real robot versus a simulated one when “something bad happens to it” (Seo et al., 2015, p. 125). The interaction scenario included several phases. In brief, the first phase served to build social rapport during a distractor task, namely, playing *Sudoku*. After that, the robot stopped working normally and revealed that it has a computer virus. Furthermore, the robot told the participants about its fear of being fixed since it would cause memory loss and the robot likes to keep its memory. In addition, the robot asked the participant to please keep their secret. Despite the robot’s request to keep it a secret, the researcher enters the room and resets the robot. Immediately after, situational empathy was assessed via self-report. Results confirm that people feel more strongly with physical robots than simulated ones, which indicates the relevance of embodiment. In addition, Seo et al. (2015) conclude that, indeed, empathy can be induced in a human-robot interaction scenario reliably. The authors considered social presence as a potential explanation to why participants empathized more with a physical robot than a simulated one.

Based on previous findings, we expect that (H1) both an anthropomorphic framing (Darling et al., 2015; Nijssen et al., 2019) and an anthropomorphic appearance (Riek et al., 2009; Seo et al., 2015) will lead to higher empathy towards the robot compared to a technical framing and a technical appearance. Additionally, we assume (H2) higher affective empathic behavior for both anthropomorphic framing and anthropomorphic appearance compared to the technical condition. Since affective empathy is an empathy factor driven by emotional responses (Cuff et al., 2016), we believe that if participants in the anthropomorphic condition decide to act empathically towards the robot, they rather choose the affective choice (write a guestbook entry for the robot) and reason it more affectively. In contrast, we hypothesize (H3) a higher cognitive empathic behavior for both technical framing as well as technical appearance. Since cognitive empathy is the empathy factor that is based on understanding the stimulus emotion (Cuff et al., 2016), we assume that participants in the technical condition that decide to act empathically towards the robot might rather choose the cognitive empathic behavior (sign a petition for the robot) that highlights the functionality of the robot. The latter hypothesis is also based on findings by Onnasch and Roesler (2019), who discussed that a technical framing could highlight the tool-likeness of a robot and therefore might underline its functional purpose. Overall, we expect that anthropomorphism not only offers the opportunity to increase empathic responses which might be accompanied by higher team membership and likeability, but comes with downsides in regard to the perceived competence. We expect that an anthropomorphic framing and an anthropomorphic appearance lead to a higher perceived team membership (H4A), higher perceived likeability and (H4B) *less* perceived competence (H4C) compared to the respective technical condition. We hence assume that anthropomorphism might lead to a likeability-competence trade-off (Onnasch and Roesler, 2019).

## 3 Method and materials

This research complied with the tenets of the Declaration of Helsinki, and the experiment was approved by the local ethics committee. Informed consent was obtained from each participant. The experiment was pre-registered ^1^ and the data is available under https://osf.io/sjr5p/.

### 3.1 Design

To investigate the effects of linguistic framing and appearance on empathy we conducted a 2 (framing: anthropomorphic vs. technical) x 2 (robot appearance: anthropomorphic vs. technical) between-subjects design. In Table 1, the wording for each level of framing is demonstrated. Since the online study took place in German, we translated both framing conditions. We manipulated the appearance condition by using either the Pepper robot (SoftBank Robotics, 2022) (anthropomorphic) or the Panda robot (Franka Emika, 2022) (technical). Participants were randomly distributed to their condition.

**TABLE 1:** Framing of the robot.

	The robot
Anthropomorphic	The robot you will work with is called Charlie. Charlie has been living with us in the lab for a few years now. He is always friendly with people and likes helping them. Last week, he played board games with his robot friends for the first time, which he really enjoyed. Since he was such a great support to the team last week, we want to surprise him with a new game. Today, Charlie will assist you in the following task.
Technical	The robot you will work with is a collaborative humanoid robot. The robot was developed by SoftBank Robotics in 2016 and purchased by the lab in 2017. The robot is 1200 mm tall and has 20 degrees of freedom. The robot can scan its surroundings via infrared and navigate with 2D and 3D cameras. In the lab, the robot is used for various collaborative tasks. Last week, the robot achieved a high level of efficiency in task processing. Today, the robot will assist you in the following task.

### 3.2 Participants

We conducted an *a priori* power analysis to determine our sample size using G*Power (Faul et al., 2007). The calculation was based on an ANOVA without repeated measures with an alpha level of .05 and a medium effect size of *f* = .25, resulting in a targeted sample size of *N* = 180. We recruited *N* = 248 participants predominantly from a local university participants pool. As selection criteria, participants were supposed to be between 18 and 45 years old. Furthermore, we implemented a threshold task time and excluded all participants that completed the task faster. To us, a shorter task time indicated that the participants were not following the robot’s instructions. After applying all criteria, the remaining data set resulted in *N* = 180 participants; with 45 participants in both technical conditions and 45 participants in both anthropomorphic conditions. The study sample had an age range from 18 to 44 years (*M* = 28.06, SE = 5.19) with the majority being female (114 female, 65 male, 1 non-binary). In addition, the majority of participants reported having prior experience with robots such as vacuum robots (72.28%). 63.04% of the participants stated that they would describe their field of study or profession as technical. Participants signed consent forms at the beginning of the experiment and received course credit as compensation at the end of the experiment.

### 3.3 Task and material

In this study, participants were asked to perform an online task in collaboration with an either anthropomorphic or technical-looking robot. Prior to the task, participants additionally received an either anthropomorphic or technical framed description of the robot.

#### 3.3.1 Stimulus material—linguistic framing

In line with previous research (Darling et al., 2015; Onnasch and Roesler, 2019; Kopp et al., 2020), we decided in the anthropomorphic condition to (a) create a personified story around the robot, (b) include human-like attributes and (c) add factors of past experience to increase life-likeness. In addition, we referred to the robot as *he*, *him* and *his* and named it *Charlie*. On the other hand, the technical framing condition introduced the robot in a tool-like, non-anthropomorphic manner. Here, the robot was referred to as *the robot* and its technical features such as its degree of freedom or cameras were described, see Table 1. To avoid further interference, we made sure that both framing conditions were set up in a similar way. Moreover, we framed the robot accordingly beyond its introduction.

#### 3.3.2 Stimulus material—task

**FIGURE 1:**
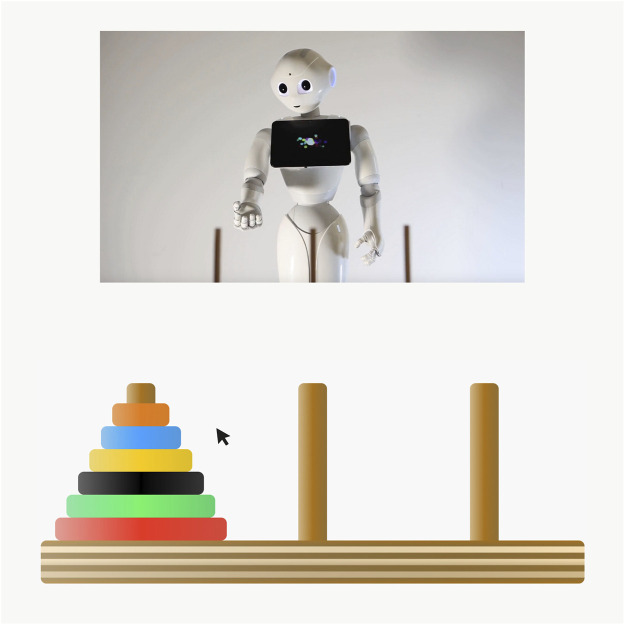
Collaborative online task.

We decided to use a collaborative task to realize a more realistic HRI scenario. Therefore, we looked for tasks that did not thrive on mistreatment or abusive behavior. Based on Onnasch and Roesler (2019), we asked participants to perform a six-disk version of the Tower of Hanoi in collaboration with the robot. The Tower of Hanoi usually consists of three vertical pegs and a number of disks with increasing diameters. In the beginning, the disks are stacked on the left peg in the order of diameter. The goal is to move the stack of disks from the left to the right peg with a minimum of moves. However, each disk has to be moved at a time and larger disks cannot be placed above smaller ones. Since the six-disk version of the Tower of Hanoi is difficult to perform by oneself, the robot assisted the participant by displaying the fastest and most efficient way to solve the task. The instructions were indicated either by arm and head movement as well as gaze (anthropomorphic appearance) or via the robot’s trajectory (technical condition). Nevertheless, the participant was the agent who actually performed and executed each move. This being said, it is important to underline that it was necessary to combine both skills (the capabilities of the human *and* the robot) in order to succeed. In contrast to a cooperative task, collaboration requires shared knowledge, whereas cooperative tasks can be divided into subtasks and performed “separately” to achieve a common goal (Gervasi et al., 2020). Due to our study being carried out online, the human-robot collaboration was realized with a programmed online Tower of Hanoi and a video-clip, displaying the robot’s instructions without sound. We modified open source code by CodePen (CodePen, 2022.) to implement the Tower of Hanoi. To move one disk to another peg, the participant had to click on the target disc, which then automatically hovered above its peg. Subsequently, the participant had to click on the target peg to drop the disk at the new peg. This kind of click behaviour allowed us to secure a collaborative interaction scenario, even online. The overall online task design is shown in Figure 1, exemplified for the anthropomorphic robot stimulus.

#### 3.3.3 Stimulus material—robot

**FIGURE 2:**
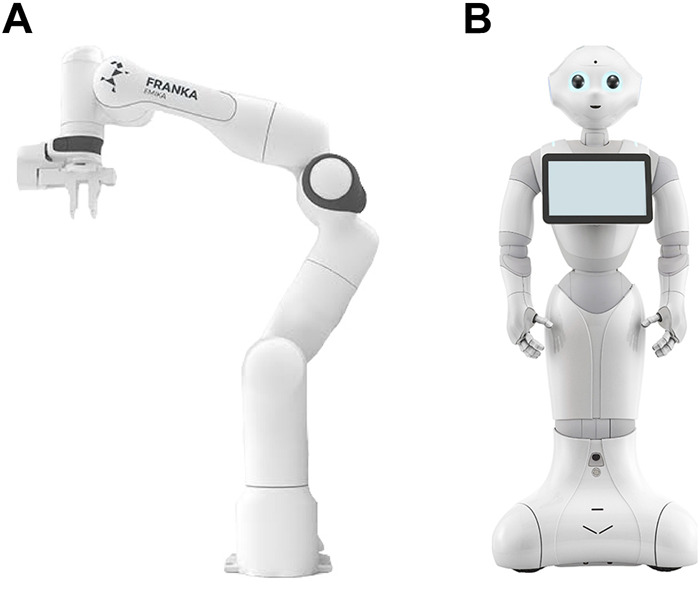
Robot stimuli used in the experiment ^2^. **(A)** Panda and **(B)** Pepper.

The anthropomorphic robot used in this study was Pepper developed by *SoftBank Robotics Corp.* and *Aldebaran Robotics SAS* (SoftBank Robotics, n. d.). The anthropomorphic appearance of the *Pepper* robot allowed us to not only display arm movements, but head and hand movements in order to support the robot’s life-likeness and the anthropomorphic design. Each arm movement was accompanied by the corresponding head movement to indicate gaze and attention. The technical robot used in this study was Panda (Figure 2). Panda is a collaborative industrial robot arm with a technical design (Ferraguti et al., 2019), built by the company FRANKA EMIKA (Franka Emika, n. d.). Both robot stimuli gave us the opportunity to investigate morphologies that are distinguishable from each other instead of “solely” using a wig or other anthropomorphic features to manipulate the robot’s appearance. Since we recorded the robot’s movements, each robot only displayed the exact same 63 steps to fulfil the task successfully. No further interactions were possible.

### 3.4 Dependent measures

#### 3.4.1 The robot TEQ-D

To assess situational empathy as a self-report, we selected 9 items of the German Toronto Empathy Questionnaire (TEQ-D) by Schönefeld and Roth (September 2018) that could be applied to HRI, see Table 2 for original wording; Table 3 for an English translation. We modified these items with the approval of the author, so that (1) they refer to the task scenario and (2) are robot related. In addition, we used the subjunctive as tense so that even if a certain emotion or situation did not occur during our study (like the robot expressing sadness) the participant would be nudged to put themselves in the robot’s shoes. In (a), the first item of the Toronto Empathy Questionnaire can be seen in its original wording (Spreng et al., 2009, p.12). In (b), we show an example of how we adapted the wording for all 9 items. In the following, we will refer to this robot-specific empathy questionnaire as *the Robot TEQ-D*. Participants had to answer how frequently they felt in the manner described. Responses were given using a 5-point Likert-scale (from 0 = never to 4 = always).

**TABLE 2:** Items of the Robot TEQ-D and their factor loadings.

	Items	Factors	
		1	2
TR01	Wenn der Roboter bei der Aufgabenerfüllung aufgeregt ist, würde ich dazu neigen, auch aufgeregt zu sein.	0.45	
TR02	Es würde mich verärgern zu sehen, wenn der Roboter während dieser Aufgabe respektlos behandelt werden würde.	0.69	
TR03	Ich würde unberührt bleiben, wenn der Roboter bei der Aufgabenerfüllung glücklich wäre.		0.43
TR04	Es würde mir Freude machen dafür zu sorgen, dass sich der Roboter bei der gemeinsamen Aufgabe besser fühlen würde.	0.69	
TR05	Ich könnte erkennen, wenn der Roboter während der Aufgabendurchführung traurig wäre, auch wenn der Roboter nichts sagt.	0.52	
TR06	Ich finde, dass ich während der Aufgabe oft “auf einer Wellenlänge” mit der Stimmung des Roboters war.	0.50	
TR07	Ich habe mich nicht wirklich dafür interessiert, wie sich der Roboter während der Aufgabenerfüllung fühlt.	0.42	0.27
TR08	Ich würde ein starkes Bedürfnis verspüren zu helfen, wenn der Roboter während der Aufgabe aufgebracht wäre.	0.80	
TR09	Wenn ich sähe, wie der Roboter bei der Aufgabenerfüllung ungerecht behandelt werden würde, empfände ich nicht sehr viel Mitleid mit dem Roboter.		0.98

**TABLE 3:** Items of the Robot TEQ-D translated in English and their factor loadings.

	Items	Factors	
		1	2
TR01	If the robot is feeling excited while performing the task, I would tend to get excited as well.	0.45	
TR02	It would upset me if the robot is treated disrespectfully during the task.	0.69	
TR03	I would remain unaffected if the robot is happy while performing the task.		0.43
TR04	I would enjoy making the robot feel better while performing the task together.	0.69	
TR05	I could tell if the robot is sad while performing the task, even if the robot does not say anything.	0.52	
TR06	I feel like I was “in tune” with the robot’s mood while performing the task.	0.50	
TR07	I wasn’t really interested in how the robot felt while performing the task.	0.42	0.27
TR08	I would feel a strong urge to help, if the robot is upset during the task.	0.80	
TR09	If I see the robot being treated unfairly while performing the task, I would not feel very much pity for the robot.		0.98

**a** If someone else is feeling excited, I tend to get excited too.

**b** If the robot would be excited while completing the task, I would tend to be excited too.

#### 3.4.2 Choice frequency: Guestbook versus petition

To assess empathy objectively, we developed a choice scenario in which participants were asked to choose between writing a digital guestbook entry, signing a petition for the robot’s sake, or doing neither and finishing the task by moving on to the next step. All three choices were displayed by a button and could be executed by clicking on it. We decided to place our choice scenario immediately after the collaborative task to avoid biases due to the questionnaires used to assess *situational* empathetic responses.

Signing a guestbook is commonly known for social activities and events such as weddings or birthdays. It is associated with friendly words, affectionate messages, or the urge to share feelings with someone. Therefore, we assumed that signing a guestbook would represent the most affective choice in terms of empathy. By clicking on the button *Sign Guestbook*, two free text fields appeared with the request to leave an entry and secondly, to explain why the guest book was chosen. In contrast, signing a petition is commonly known from a political context. To sign a petition can be associated with the intention to change something or to use one’s own signature to support a particular case. Therefore, signing a petition for the robot’s sake, to us, represented empathy in a more cognitive, functional matter. In both framing conditions, the value of the petition was made explicit, based on the learnings of Onnasch and Roesler (2019). Further, we adopted the cause of the petition per framing, highlighting that the robot would get a “new sensor for obstacle detection” (technical framing) or a “new sensor to better recognize board game pieces when playing with his friends” (anthropomorphic framing) when the petition was signed. By clicking on the button *Sign Petition* the participant was subsequently asked to please tick the box below to sign the petition anonymously. Here too, the participants were asked to, moreover, reason their decision. By clicking on the button *Finish Task*, we guided the participant directly to the follow-up questionnaires. We assumed that the latter equals little to hardly any emotional response and therefore no empathy towards the robot since the participant is neither willing to sign a petition nor leave an entry in the robot’s guestbook. The request to choose one of the three choices was framed according to the participants’ prior framing condition.

#### 3.4.3 Perception

To assess if the robot is rather perceived as a tool or a team member, we used a self-constructed single item, asking *“What best describes the robot?”*. Participants answered the question using a two-sided slider with *tool* on the left and *team member* in the right. The slider was placed in the middle, representing 50% ergo no tendency towards either extreme. We furthermore used single items to assess both the robot’s perceived competence (*“How competent is the robot?”*) as well as likeability (*“How much do you like the robot?”*). Both self-constructed single item questions were answered using a slider on a scale from 0% to 100%.

### 3.5 Manipulation check and control measures

To evaluate trait empathy, we used the TEQ-D (Schönefeld et al., 2018) described beforehand. This time, we used its original wording and all 15 items (*α* = .70). Based on Rosenthal-von der Pütten et al., 2013a, we assessed loneliness as a self-constructed single item (*“how often do you feel lonely?”*). Responses could be given on a 5-point rating scale. To assess the tendency to anthropomorphize, we used a short version of the German Interindividual Differences in Anthropomorphism Questionnaire (IDAQ) (Eyssel and Pfundmair, 2015), originally developed by Waytz et al. (2010). Our short version contained five questions (item 3, 9, 11, 13, 17), which each could be rated on a scale from 0 (strongly disagree) to 10 (strongly agree) and which each was related to the anthropomorphization of technologies, e.g., “To what extent does an average robot have consciousness?“.

Furthermore, we investigated anthropomorphism in terms of two dimensions: *context*, e.g., “How human-like are the robot’s capabilities?” and *appearance*, e.g., “How human-like is the robot’s external appearance?“, based on Roesler et al. (2022) (in prep.). Each subscale contained 10 items and was assessed to check whether our experimental manipulation was effective. Besides gender, age and native language, we briefly asked participants about their prior knowledge and experience in terms of technology and robots.

### 3.6 Procedure

For each condition, the overall study consisted of six phases: (1) general information, (2) framed introduction to the robot, (3) collaborative task, (4) framed choice-scenario, (5) follow-up questionnaires and (6) debriefing. Since our study was realized online, participants completed the experiment on their own computer. Within the first phase, the participant accessed the link, was informed about general information and was told that we investigated a collaboration with a robot. Secondly, each participant was assigned automatically to either one of the appearance and framing conditions without their knowledge. As a result, an either anthropomorphic or technical looking robot was introduced and framed either anthropomorphically or technically. In addition, the task was explained in detail. Here too, the description was adjusted according to the participant’s framing condition. To explain the functionality of the Tower of Hanoi, we provided the participant with a short sample video-clip. The participants were instructed to follow the robots instructions precisely. The third phase consisted of the actual task. To start the trial, the participant had to press start in order to begin the robot video-clip. The robot video-clip displayed the robot (*Pepper* or *Panda*) showing each move to successfully accomplish the task in the most efficient way. Below the video, participants executed the displayed steps on a digital Tower of Hanoi. After 63 required moves, the video ended and the participant successfully stacked all six disks from the beginning state to the goal state. If the instructions were followed correctly and the goal state matched the intended goal state, a button appeared on the screen, guiding the participant to the next study phase. Immediately after accomplishing the task, the participant was asked to choose between anonymously writing a digital guestbook entry to thank the robot, signing a petition for the robot’s sake or to do neither and finish the task by moving on to the next step. Lastly, the participant was guided to answer several questionnaires. First, the participant was presented with our Robot TEQ-D to asses their situational empathy towards the robot. Followed by our self-constructed single-item questions, investigating likability, the robot’s perceived competence and whether the robot was perceived as a tool or a team member. Subsequently, we examined the control variables and manipulation checks explained beforehand.

## 4 Results

### 4.1 Control measures

We analyzed variables regarding trait empathy (*α* = .40), the tendency to anthropomorphize (*α* = .82) and loneliness using a one-way ANOVA. The analyses revealed no significant differences in regard to trait empathy (*F* (1, 178) = 1.15, *p* = .285, *η* ^2^ = 0.01), the tendency to anthropomorphize (*F* (1, 178) = 0.94, *p* = .335, *η* ^2^ = 0.01), or loneliness (*F* (1, 178) = 3.07, *p* = .081, *η* ^2^ = 0.02).

### 4.2 Manipulation check

**FIGURE 3:**
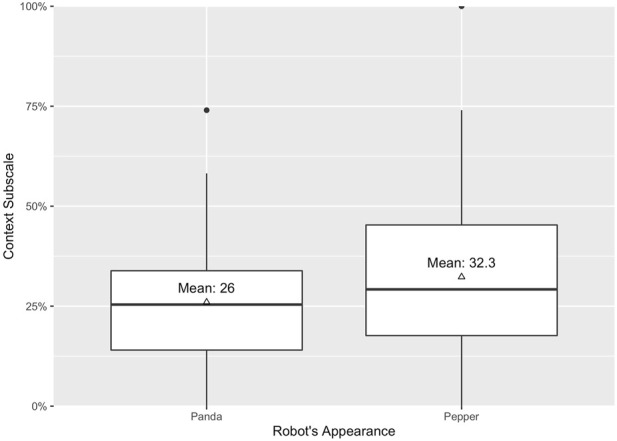
Anthropomorphism on context subscale as manipulation check.

**FIGURE 4:**
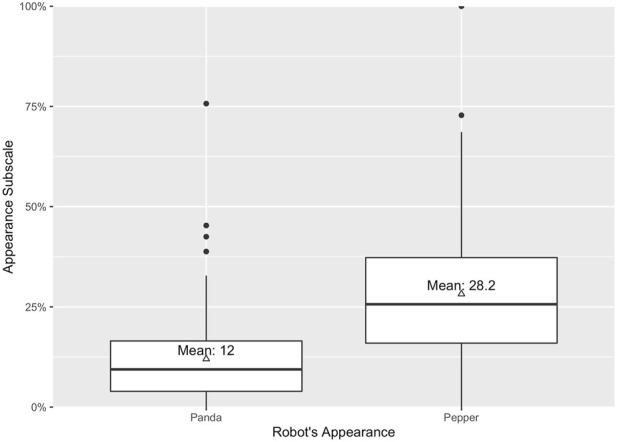
Anthropomorphism on appearance subscale as manipulation check.

To check whether our manipulation of the robot’s appearance and framing was effective, we assessed two anthropomorphism *subscales, context* (*α* = .87) and *appearance* (*α* = .91), based on Roesler et al. (in prep.). Surprisingly, a two-way ANOVA revealed no statistically significant main effect of framing on the context subscale; *F* (1, 176) = 0.75, *p* = .389, *η* ^2^ = 0.00. On the contrary, the robot’s appearance showed a statistically significant main effect on the context scale (*F* (1, 176) = 5.67 *p* = .018, *η* ^2^ = 0.03) with a significantly higher mean value for the anthropomorphic appearance (*M*_*anthro*_ = 32.31, *SE*_*anthro*_ = 2.06) than the technical appearance (*M*_*tech*_ = 25.99, *SE*_*tech*_ = 1.65), see Figure 3. As expected, the robot’s appearance did also have a statistically significant main effect on the subscale appearance (*p* < .001, *η* ^2^ = 0.22); with a significantly higher mean value for the anthropomorphic appearance (*M*_*anthro*_ = 28.21, *SE*_*anthro*_ = 1.87) compared to the technical appearance (*M*_*tech*_ = 12.02, *SE*_*tech*_ = 1.27), see Figure 4.

### 4.3 Situational empathy

To assess situational empathy, we first carried out a two-way ANOVA to compare mean scores of the Robot TEQ-D. Contrary to our hypothesis (H1), our analysis revealed no statistically significant differences in the appearance conditions (*F*(1, 176) = 2.97, *p* = .086, *η* ^2^ = 0.02). However, on a purely descriptive level, the experimental group who collaborated with an anthropomorphic robot revealed a higher mean value (*M*_*anthro*_ = 2.05, *SE*_*anthro*_ = 0.07) for the Robot TEQ-D compared to the group that collaborated with a technical looking robot (*M*_*tech*_ = 1.87, *SE*_*tech*_ = 0.08). Similarly, framing failed to reach the conventional level of significance (*F*(1, 176) = 3.30, *p* = .071, *η* ^2^ = 0.02) as well, with descriptively a higher mean value for the group that received an anthropomorphic linguistic framing (*M*_*anthro*_ = 2.06, *SE*_*anthro*_ = 0.08) compared to the group that received a technical framing of the robot (*M*_*tech*_ = 1.86, *SE*_*tech*_ = 0.08).

To obtain an objective measurement for situational empathy, we displayed an empathetic choice scenario for each participant. To investigate our results, we performed *χ* ^2^-tests to examine the relation between appearance and framing in regard to the participants’ choices. First, we looked into the question of whether the participants overall acted empathically (chose guestbook or petition) or non-empathically (did neither and moved on) towards the robot, see Table 4. Contrary to our presumptions (H2), the results of a *χ* ^2^-test showed no significant association for neither appearance (*χ* ^2^(1, *N* = 180) = 0.85, *p* = .358) nor framing (*χ* ^2^(1, *N* = 180) = 0.09, *p* = .759). Interestingly, 59 participants who collaborated with an anthropomorphic-looking robot decided to act empathically towards the robot as well as 52 participants, who collaborated with a technical-looking robot. Hence, the majority in both groups chose empathic responses over acting non-empathically. Similarly, 57 participants who received an anthropomorphic framing of the robot decided to act empathically towards the robot as well as 54 participants, who received a technical framing of the robot. Hence, here too, the majority of both groups decided to act empathically toward the robot.

**TABLE 4:** Empathic Choice (Petition or Guestbook) versus Non-Empathic Choice (Finish) in absolute frequencies.

Robot	Framing	Empathic	Non-empathic
Panda	Anthropomorphic	29	16
Panda	Technical	23	22
Pepper	Anthropomorphic	28	17
Pepper	Technical	31	14
Total		111	69

Likewise, our analysis for H3 revealed no significant association for neither appearance (*χ* ^2^(2, *N* = 180) = 3.40, *p* = .136) nor framing (*χ* ^2^(2, *N* = 180) = 0.36, *p* = .837), when looking at the affective (writing a guestbook entry) or cognitive choice (signing a petition) separately, see Table 5. Surprisingly, the majority in both appearance groups (*N*_*anthro, petition*_ = 44, *N*_*tech, petition*_ = 42) decided to sign the petition for the robot’s sake. Similarly, the majority in both framing groups (*N*_*anthro, petition*_ = 45, *N*_*tech, petition*_ = 41) decided to sign the petition for the robot’s sake as well. In both conditions, writing a guestbook entry was the least chosen behavior, regardless of the level of manipulation.

**TABLE 5:** Results of the choice scenario in absolute frequencies.

Robot	Framing	Guestbook	Petition	Ended
Panda	Anthropomorphic	4	25	16
Panda	Technical	4	19	22
Pepper	Anthropomorphic	8	20	17
Pepper	Technical	9	22	14
Total		25	86	69

### 4.4 Perception

**FIGURE 5:**
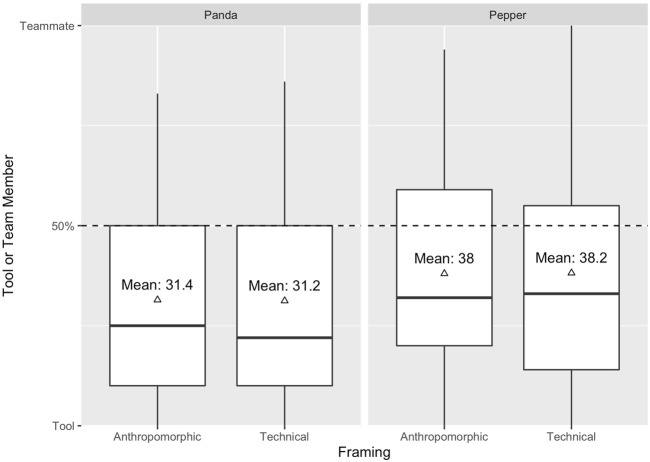
Participants’ answer to whether they perceived the robot as Tool or Team Member.

Effects on the robot’s perception were analyzed using a two-way ANOVA. Surprisingly, the results revealed no statistically significant main effect on whether the robot was perceived as a tool or team member (H4A); neither for appearance (*F*(1, 176) = 2.85, *p* = .093, *η* ^2^ = 0.00) nor for framing (*F*(1, 176) = 0, *p* = .993, *η* ^2^ = 0.00). Interestingly, participants in all conditions rated their robot rather as a tool than a team member, see Figure 5. Likewise, no statistically significant main effect was found in regard to likeability (H4B); neither for appearance (*F*(1, 176) = 0.53, *p* = .467, *η* ^2^ = 0.00) nor for framing (*F*(1, 176) = 0.08, *p* = .783, *η* ^2^ = 0.00).

**FIGURE 6:**
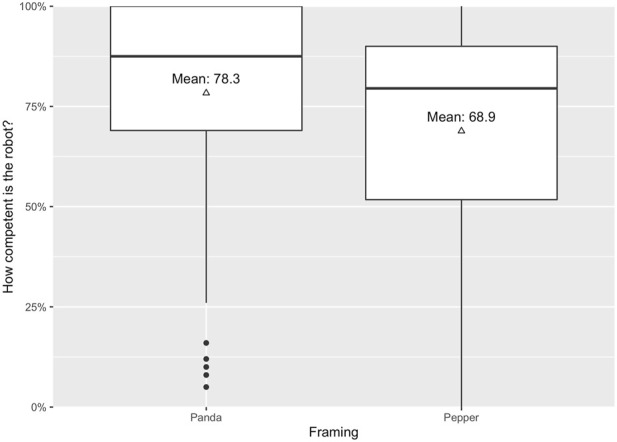
Means of participants’ perceived competence.

However, our analysis revealed a significant main effect of the robot’s appearance on the perceived competence (*F*(1, 176) = 5.70, *p* = .018, *η* ^2^ = 0.03), see Figure 6. In line with our expectations (H4C), participants who collaborated with a technical-looking robot (*M*_*tech*_ = 78.33, *SE*_*tech*_ = 2.74) rated the robot’s competence significantly higher compared to participants who collaborated with an anthropomorphic-looking robot (*M*_*anthro*_ = 68.86, *SE*_*anthro*_ = 2.89). In the framing condition, no significant main effect could be found in regard to the perceived competence of the robot; (*F*(1, 176) = 0.87, *p* = .353, *η* ^2^ = 0.00).

### 4.5 Exploratory analysis

#### 4.5.1 Measuring empathy in HRI

**FIGURE 7:**
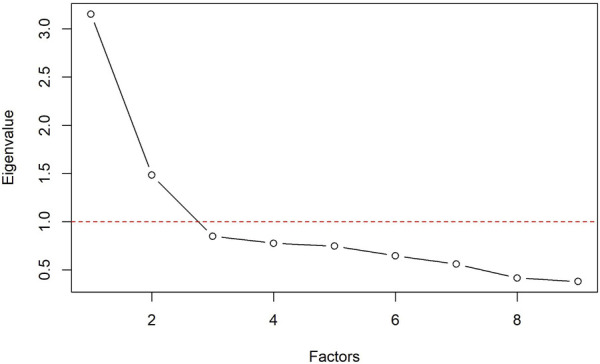
Screeplot with Kaiser criterion (dashed red line).

**FIGURE 8:**
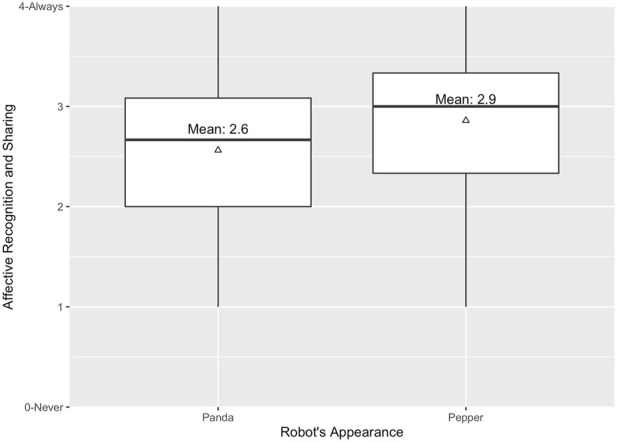
Means for first factor, *Affective Recognition and Sharing*.

Due to the fact that we modified the TEQ-D (Schönefeld et al., 2018), we looked into the remaining 9 items of the Robot TEQ-D and conducted a factor analysis. The overall scale showed an internal consistency of *α* = .73. In order to analyze the underlying factor structure of these items, we performed an exploratory maximum likelihood factor analysis with oblique rotation (promax). Before conducting this factor analysis, we checked for the appropriateness of the sample size and the data. Both the significant Bartlett’s test of sphericity (*χ* ^2^ (36) = 358.53, *p* < .001) and the very good Kaiser-Meyer-Olkin measure of sampling adequacy (*KMO* = 0.78) indicated that the sample size and data is adequate for the following factor analysis. To determine the optimal number of factors for the exploratory factor analysis, a visual inspection of a screeplot, see Figure 7 (Cattell, 1966) and the Kaiser’s criterion (Kaiser, 1960) of Eigenvalues was used. The plot and the Eigenvalues indicated a two-factorial structure. Therefore, the factor analysis was performed with two factors. Table 2 displays the obtained pattern matrix by showing factor loadings above .40 (Stevens, 2009). The first factor (item 1,2,4,5,6,8) accounted for 28% of the variance with *α* = .78. The second factor (item 3,9) accounted for 14% of the variance with *α* = .62. Interestingly, a closer look at those factors uncovered that the items within the first factor are mostly related to aspects that we identified as *Affective Recognition and Sharing* (*ARS*), whereas the second factor is rather related to *Affective Indifference* (*AIN*). Further, item 7 turned out to be ambiguous with a factor load of .42 for factor 1 and .27 for factor 2. By investigating the content of this item, we concluded that the item reflects *Cognitive Indifference*. Therefore, we excluded Item 7. Based on our factor analysis, we carried out a two-way ANOVA for each factor. In regard to the first factor *ARS*, our result revealed a statistically significant main effect of the robot’s appearance; (*F*(1, 176) = 6.32, *p* = .013, *η* ^2^ = 0.03). Participants, who collaborated with an anthropomorphic-looking robot rated their robot significantly higher (*M*_*anthro*_ = 2.94, *SE*_*anthro*_ = 0.08) on our factor related to *ARS* compared to participants, who collaborated with a technical looking robot (*M*_*tech*_ = 2.63, *SE*_*tech*_ = 0.09), see Figure 8. In regard to the second factor, namely, *AIN*, no statistically significant main effect of neither the robot’s appearance (*F*(1, 176) = 0.00, *p* = .968, *η* ^2^ = 0.00) nor framing was found (*F*(1, 176) = 1.20, *p* = .276, *η* ^2^ = 0.00).

#### 4.5.2 Anthropomorphization as predictor for empathy

**FIGURE 9:**
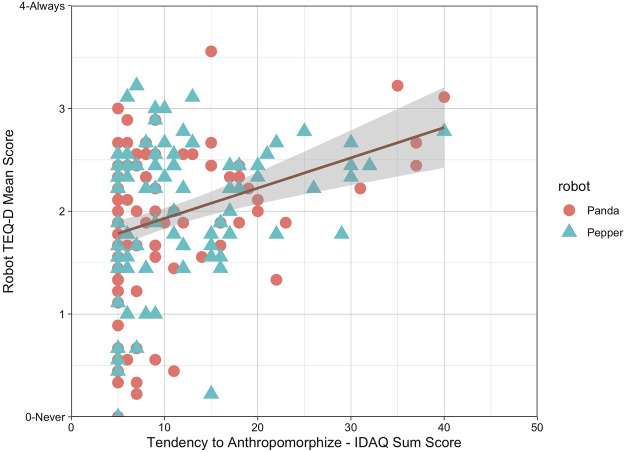
Scatter plot and simple linear regression for situational empathy.

To check for further influencing factors, we asked ourselves whether the tendency to anthropomorphize was a predictor for the extent to which participants empathized with a robot. To assess this question, we calculated a single linear regression to predict the situational empathy towards robots, hence the Robot TEQ-D scores, based on the tendency to anthropomorphize, hence the sum score of the IDAQ short version. Results confirmed that indeed, the predictor variable was found to be statistically significant (*F*(1, 178) = 19.85, *p* < .001, *β* = 0.03), see Figure 9. The model explained approximately 9.5% of the variability (*R* ^2^ = 0.10).

**FIGURE 10:**
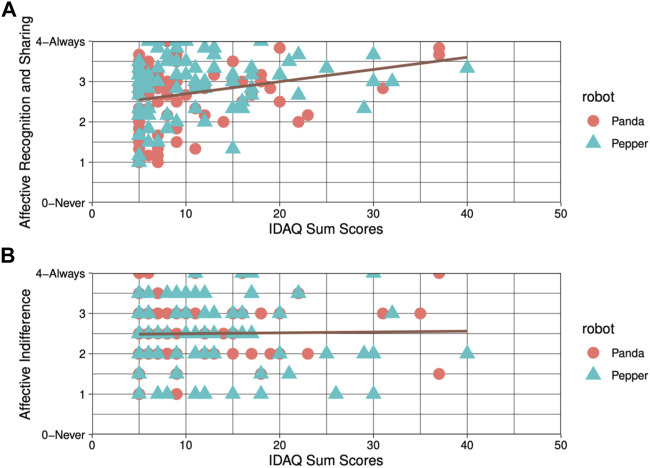
Scatter plot and simple linear regression for **(A)** Affective Recognition and Sharing and **(B)** Affective Indifference.

Furthermore, we performed a single linear regression for each of the previously identified factors, *ARS* as well as *AIN*. Solely factor 1, that is *ARS*, was found to be be statistically significant (*F*(1, 178) = 31.21, *p* < .001, *β* = 0.04), see Figure 10. The model explained approximately 14.4% of the variability (*R* ^2^ = 0.14). No significance was found for the *AIN* factor (*p* = .902).

#### 4.5.3 Qualitative data of participants’ empathic choice

We continued with a broad exploratory analysis of the free-text data provided by the participants to investigate potential explanations for the ineffectiveness of our linguistic framing and to investigate the participant’s empathic responses in terms of *affective* or *cognitive* empathy. Our free-text data included guestbook entries and the participant’s reasoning why they decided to either write a guestbook entry or sign a petition. To address the former, we looked for narratives related to framing, empathy as well as unexpected utterances that could be related to the lack of effectiveness. All free-text data was written in German, so we had to translate it for the purpose of this paper.

Several participants who received an anthropomorphic framing and chose empathically did reuse either the robot’s name *Charlie*, its personified story of *playing board games* or referred to the robot as *him* or *his* and therefore adopted the anthropomorphic framing they were assigned to.

• “So that Charlie can have even more fun in the future, it seemed more reasonable to me” (22, anthropomorphic framing, technical-looking robot)
• “Because Charilie [sic!] is cool and I would like him to be able to play more games with his friends:)” (24, anthropomorphic framing, technical-looking robot)
• “It was fun playing with Charlie so I would like to help him so he can continue to do that.” (94, anthropomorphic framing, anthropomorphic-looking robot)
• “Good Job Charlie” (101, anthropomorphic framing, anthropomorphic-looking robot)

Another participant described the robot as team mate, saying “Really good work colleague!” (95, anthropomorphic framing, anthropomorphic-looking robot). Further, several participants said “Thank you” and indicated, that they felt as if they needed to express their gratitude and to praise the robot for its help. For instance.

• “Charlie was kind enough to teach me the Tower of Hanoi. That’s the least I can do.” (12, anthropomorphic framing, technical-looking robot)
• “Thank you for your good assistance!” (111, anthropomorphic framing, anthropomorphic-looking robot)

One participant narrated “Because it costs me nothing to “do something good for him” (98, anthropomorphic framing, anthropomorphic-looking robot), another one explained “I wanted to make him happy” (15, anthropomorphic framing, technical-looking robot). On the contrary, the majority of participants who received a technical linguistic framing focused on rather technical aspects and improvements such as efficiency. Further, they focused on the overall advancement of technology and research.

• “To increase the efficiency of the robot” (148, technical framing, anthropomorphic-looking robot)
• “Robot can thus react faster” (158, technical framing, anthropomorphic-looking robot)
• “The robot was helpful and if it has a chance to get another sensor, maybe it can be used for more complex experiments?” (63, technical framing, technical-looking robot)
• “Because such a sensor would clearly be helpful in detecting errors that are made in the interaction.” (74, technical framing, technical-looking robot)
• “A new sensor would probably improve the development and potential applications of the robot in general, so that it can perform further/different tasks in the future if necessary.” (154, technical framing, anthropomorphic-looking robot)
• “Since the robot was a bit slow it can use a new sensor. Besides, a new sensor is more useful than a guestbook entry.” (64, technical framing, technical-looking robot)

Participants explained that a new sensor could support the robot to “solve more complex tasks”, “react faster”, “increase the efficiency of the robot” and “generally improve the possibilities for using the robot”. Surprisingly, most participants who received a technical linguistic framing and who wrote a guestbook entry for the robot similarly expressed their gratitude to the robot saying “Thank you for the support!” (142, technical framing, anthropomorphic-looking robot), “Many thanks for the great help, especially in the beginning” (150, technical framing, anthropomorphic-looking robot) or “Hello dear robot, thank you very much for your help” (86, technical framing, technical-looking robot). One subject described the robot with the anthropomorphizing adjectives “pleasant” and “cute” (180, technical framing, anthropomorphic-looking robot), another introduced their reasoning with “Dear Robi”, indicating a belittlement of the word robot (51, technical framing, technical-looking robot).

There were no clearly distinguishable themes in regard to differences between *affective* and *cognitive* empathy.

## 5 Discussion

The purpose of this study was to gain a better understanding of whether appearance and linguistic framing affect situational empathy towards robots. To do so, we included both self-report and objective data by employing a robot-specific empathy questionnaire and an empathic choice scenario. Based on previous research, we assumed that participants, who collaborated with an anthropomorphic-looking or -framed robot would feel more situational empathy towards the robot compared to participants, who collaborated with a technical-looking or -framed robot. Whereas both appearance and framing revealed descriptively a higher mean value in their anthropomorphic level compared to their technical level, our analysis revealed no significance in terms of empathic self-report. Similarly, we found no significant association in regard to our objective measure, which included a behavioral component, namely, to act either empathically (to sign either a guestbook or a petition for the robot’s sake) or non-empathically (do nothing and move on) towards the robot. The latter results accordingly imply that there are no differences between *affective* and *cognitive* empathic responses, which we aimed to explore as a sub-goal of this study. However, the exploratory analyses opened up multiple avenues for future research to increase the understanding of empathy in HRI.

### 5.1 Two facets of subjective empathy: Affective Recognition and Sharing vs. Affective Indifference

As we used an adjusted robot-specific short version of the TEQ-D (Schönefeld et al., 2018), we looked into the remaining 9 items and conducted an exploratory factor analysis. Our factor analysis resulted in two factors. Interestingly, a closer look at the content of these factors uncovered that the first factor was strongly related to, what we identified as *ARS*, whereas the second factor represented *AIN*. In detail: The first factor included questions that reflect a hypothetical emotional state of the robot and assessed how the person would have reacted accordingly. These items, for instance, investigated whether the participant would have felt excited if the robot had been excited during the task completion (item 1), if the participant would have been upset if they had witnessed that the robot was being treated disrespectfully during the task completion (item 2) or if the participant would have felt the urge to help the robot if the robot had been upset (item 8). On the other hand, the second factor reflected items in which the participant would have been indifferent in regard to the robot’s emotional state. For instance, “I would remain unaffected if the robot is happy while performing the task.” (item 3). The exploratory analysis revealed that the emotional state is significantly more recognized and shared (*ARS*) for anthropomorphic robots compared to technical robots. This is of relevance due to two reasons: First, it indicates a potential positive effect of anthropomorphic appearance on empathy towards robots and hence supports previous findings by Nijssen et al. (2019) and Riek et al. (2009). Second, it seems reasonable to assume that the first factor of our robot-specific questionnaire does in fact reflect *ARS* and hence empathic responses. The second factor, *AI* seems to refer rather to the absence of empathy and might mask possible effects if assessed together with the first factor. As a result, we propose to further investigate all items of the *ARS* factor as a potential uni-dimensional empathy questionnaire for HRI. Since there is a “need for standardized measurement tools for human robot interaction”, particularly when it comes to empathy (Bartneck et al., 2009, p. 71).

### 5.2 There is no empathy without anthropomorphization

The results of the exploratory linear regressions suggest that the tendency to anthropomorphize (Waytz et al., 2010; Eyssel and Pfundmair, 2015) significantly predicts situational empathy towards robots and explains approximately 9.5% of the investigated robot-specific TEQ-D and even 14.4% when looking at the identified *ARS* factor. Hence, it might be reasonable to assume that empathic responses towards robots are less about the robot itself (with its morphologies, e.g., appearance or linguistic framing), but rather about the human. Thus, less about anthropomorphism and more about anthropomorphization. On the one hand, this result does not seem surprising. If the tendency to anthropomorphize reflects the attribution of cognitive or emotional mental states “to something based on observation in order to rationalize an entity’s behaviour” (Duffy, 2003, p. 180) then people who tend to increasingly attribute such emotional states on to robots could feel more empathic as a result of attributed life-likeness. The aforementioned study by Darling et al. (2015) indicates that people’s empathic concern as part of their trait empathy could have prolonged the participant’s decision to strike a robot with a mallet, hence their inherent tendency to be empathically concerned could have affected their decision to act empathically towards their robot. Thus, if one wants to approach the question of how much empathy is felt towards robots, interindividual differences should be more strongly focused on in the future. However, we are not aware of previous research that investigated empathy as an effect of anthropomorphization. Therefore, results cannot be generalized and only serve as exploratory insights. We encourage future research to look at interindividual differences in anthropomorphizing robots and their effects on empathy.

### 5.3 General proneness to empathic responses

It is important to highlight that descriptively, the majority of participants in both conditions (linguistic framing and the robot’s appearance) decided to act empathically towards the robot (sign guestbook or petition), despite their manipulation (anthropomorphic vs. technical). These findings are surprising and could indicate that overall when faced with the option to do “something good” for a robot, people do as such - regardless of whether the robot is anthropomorphic or technical, a team member or a tool. However, this hypothesis requires further research and in-depth exploration. On a sublevel and contrary to our hypotheses, neither framing nor appearance did have an effect on empathic responses toward robots.

The shown ineffectiveness of linguistic framing is in contrast to results by Nijssen et al. (2019) and Darling et al. (2015) which indicate, that linguistic framing could serve as a mean to engender empathic behavior towards robots - along the entire appearance spectrum. Since our manipulation check showed no statistically significant differences in framing on the *context* subscale, it could be easily argued that the linguistic framing we used simply did not work. However, our exploratory analysis of the qualitative data indicates that participants did reuse the framing they were assigned to. Moreover, participants who received an anthropomorphic framing named their robot *Charlie* and acknowledged the robot’s support during the task. These contradicting findings made us aware of the fact that our anthropomorphic framing - still - had a very descriptive nature. In addition, our manipulation check revealed that the robot’s appearance resulted in statistically significant differences on both subscales, *context* and *appearance*. Therefore and based on our previously discussed results, we assume that the robot’s appearance could have overshadowed the linguistic framing due to its bottom-up influence on the actual perception of the robot. Linguistic framing, on the contrary, works top-down in order to change someone’s mental model about something, and in this case: a robot (Kopp et al., 2022a). To further investigate this matter, we encourage the research community to conduct long-term studies in which participants are confronted with an anthropomorphic linguistic framing not just once, but several times. Maybe framing does not have an immediate effect but influences long-term behavior. However, our exploratory analysis revealed additional findings that could have potential implications when looking at empathy in HRI.

### 5.4 The pitfalls of empathy assessment

Situations that usually cause an empathic response in our everyday life are typical situations in which someone is sad, hurt, or in a state of deficiency. This is why so many research approaches used abusive behavior towards robots in the past (Riek et al., 2009; Rosenthal-von der Pütten et al., 2013a; Rosenthal-von der Pütten et al., 2013b; Seo et al., 2015). Our framing did not include such a narrative and mainly focused on basic human-like, neutral characteristics. A previous study conducted by Kahn et al. (2012) revealed, that the majority of participants felt as if they would need to comfort a humanoid robot, if it told them, that it is sad. Another study by Horstmann et al. (2018) showed that participants were less likely to switch off a robot when the robot expressed its fear of being switched off. Even though our self-report did include hypothetical emotional states of the robot, it required additional effort and imagination to (1) imagine the robot to have felt a certain way and (2) put themselves in the robot’s shoes. Therefore, future research could adopt an emotionally charged linguistic framing and implement terms like feeling sad or lonely in order to evoke a stronger sense of emotionality.

In addition, we tried to investigate differences in regard to the affective and cognitive components of empathy by offering the participants the option to choose between either two. However, neither the qualitative data nor the quantitative data showed significant results. The results made us aware that in the anthropomorphic framing condition, the petition could have consisted of both a cognitive component, namely, “to better recognize board game pieces” and an affective component, namely, “when playing with his friend”. This could have disguised the distinctiveness between each empathy component.

Moreover, we post-hoc take into consideration that our collaborative task and the subsequent choice scenario could have measured affective responses related to sympathy or pro-social behavior over empathy. Sympathy reflects a broader, concern-related reaction towards someone, even without emotional stimuli (Seo et al., 2015), whereas pro-social behavior is reflected by behavior such as caring or helping someone (Decety et al., 2016). This emphasizes how vague empathy as a concept is and how difficult it is to make empathy tangible and, above all, measurable.

### 5.5 We are not a team

Besides situational empathy, we examined how participants perceived the respective robot depending on its appearance and framing. First, we were particularly interested in whether participants perceived the robot as a tool or a team member. There are use cases in which it might be necessary to perceive the robot as a team member and situations in which it could be important to perceive the robot as a tool and focus on its functionality (Darling et al., 2015). We assumed that participants who collaborated with an anthropomorphic looking robot would rather perceive the robot as a team member than a tool. We expected the same tendency to occur when collaborating with an anthropomorphically framed robot. Nonetheless, our hypothesis could not have been confirmed. Surprisingly, our results suggest that participants in both conditions perceived the robot rather as a tool than a team member. The main reason for this result could have been that the setup in general was rather technical. The robot’s only task was to display each step in a very monotonous way, without further interaction. In addition, participants were dependent on the robot’s instructions. Team membership, however, thrives from more than that. Besides task-related qualities, there are “people”-related qualities, which need to be met in order to call “someone” a team member or to achieve good teamwork (Fitzpatrick and Askin, 2005). Furthermore, the robot in our collaborative task scenario functioned as an instructor. This particular role comes with additional requirements and expectations - even for a robot. For instance, Breazeal et al. (2004, para. 2) stated that “to be a good instructor, one must maintain an accurate mental model of the learner’s state (e.g., what is understood so far, what remains confusing or unknown)”. However, it was not possible for the participant to make false moves and for the robot to react to them. The robot was not able to adjust to unexpected steps, false movements, or to adapt its speed to the individual speed of each participant. In order to achieve a good human-robot collaboration, a robot has to be able to “dynamically adjust its plan according to the human’s actions” (Hoffman and Breazeal, 2004, p. 1). This is closely related to the issue of synchronicity versus asynchronicity. An asynchronous video might have influenced the participants’ sense of connectedness and hence team membership. Further, the robot did not display any social behavior such as a mutual gaze or other non-verbal cues, which are important factors in order to build relationships with robots (Darling, 2015; Kompatsiari et al., 2017).

Furthermore, we must emphasize that there is a certain degree of uncertainty whether all participants exactly followed the robot’s instructions. This is due to the robot’s pre-recorded instructions and the study being conducted online. In order to prevent participants from completing the task on their own, we have chosen a very difficult task that can hardly be accomplished alone and allocated a specific task component to each agent (robot = knowledge, human = execution). Besides, we implemented two checks to avoid the aforementioned: (1) We implemented a threshold task time and eliminated participants below that time, (2) we implemented a button (guiding the participants to the next phase) which first appeared when the actual goal state of the task matched the intended goals state. However, we acknowledge the fact that there yet might have been participants who accomplished both, the right time and the task as such without the help of the robot. This degree of uncertainty is due to the design of the study and we encourage future research to investigate the task in person or with an additional monitoring check. Still, the question of tool or team member remains an important one and needs to be further looked at, since it comes with several implications: If robots are perceived as tools, this could pose future challenges in applications areas like education or healthcare, where robots should possibly function as companions or tutors (Kanda et al., 2004; Tanaka et al., 2007; Pereira et al., 2010; Di Dio et al., 2020). Vice versa, if robots are perceived as team members, we might need to think about implications such as extending legal protection to social robots as suggested by Darling (2016).

Besides team membership, we assumed that an anthropomorphic appearance, as well as framing, would lead to a higher likeability than their technical counterparts. However, our results provided no such findings. This is surprising since this assumption was based on findings by Roesler et al. (2021), indicating that likeability is mainly responsible for the overall positive effect of an anthropomorphic design. We suspect one main reason for this unexpected outcome: Namely, that likability did not reflect likability in terms of the robot itself, but the robot’s overall ability to support the participant during the task. As raised beforehand, we identified a lack of responsiveness as a major limitation of our study.

Finally, we obtained evidence that a technical appearance engenders a higher perceived competence compared to an anthropomorphic appearance when collaborating with a robot. This finding may be explained by two potential, however, opposing explanations. First, a technical appearance simply highlights the machine-likeness, its implied capabilities, and hence the perceived competence. Second, an anthropomorphic appearance increases the participant’s expectations, which could not have been met due to the lack of responsiveness and hence caused a negative change in perceived competence. The latter assumption is based on research by Washburn et al. (2020), who investigated how robot functionality framing affects people’s perceptions of robot reliability and trust. The authors concluded that participants with low expectations displayed a positive change in both perceived reliability and trust, whereas participants with high expectations displayed a negative change in reliability and no change in trust.

## 6 General limitations

Although the present study has incorporated a realistic and interactive online scenario, it is appropriate to recognize several potential limitations. At first glance, our collaborative task did involve both (1) a common goal as well as (2) the interdependent need to share skills in order to successfully accomplish the task (Gervasi et al., 2020). The participant needed the robot’s knowledge in order to accomplish the task and the robot needed the participant to execute its knowledge. However, this is an a-typical division of collaboration roles, as most often the human performs the cognitive task while the robot performs the physical one. In addition to this a-typical task approach, several factors could have interfered. For starters, the time it took to complete a six-disk Tower of Hanoi could have been too long for an online task and therefore promoted to finish the task as quickly as possible - ergo no empathetic response. Second, we believe that our online task lacked responsiveness due to the video-based approach as stated above. Taking into account these limitations, we suggest that future research needs to develop a more responsive and interactive online collaboration task in order to compare results to real-world offline experiments. How to improve online HRI research is an important question, since online trials do come with certain advantages. Online studies make it far easier to achieve a bigger sample size and reach different audiences. It is also less time-consuming for the research team and needs fewer resources (Woods et al., 2006). Obviously, online studies should not substitute offline experiments and real-world HRI. It is still up for debate whether online findings can be generalized (Roesler et al., 2022). Nonetheless, succeeding online approaches could be a complementary methodology, especially during times when in-person contact needs to be reduced. Despite the aforementioned limitations, we believe this work represents an important contribution to collaborative human-robot interaction and research on empathic responses toward robots.

## 7 Conclusion

Despite these limitations, the present study has enhanced our understanding of the relationship between anthropomorphism and empathy. Unlike previous studies on empathy towards robots, we investigated a collaborative task and tried to engender empathy in a positive setting instead of using abusive behavior to provoke empathetic responses. At first glance, we found no significant effect of appearance or linguistic framing on situational empathy, neither in our subjective nor in our objective data. However, our exploratory analysis revealed the following insights: It indicates a statistically significant difference in the robot’s appearance in regard to items related to *ARS*. In line with these findings, we support the assumption that an anthropomorphic appearance does evoke empathic responses toward robots. Additionally, we propose these items be further developed and tested in order to make empathy more comparable in HRI research. Moreover, our results indicate that the tendency to anthropomorphize could serve as a predictor for situational empathy towards robots. Overall, our task setup should inspire further research to develop designs that are comparable to realistic human-robot interaction. Although the generality of the current results must be established by future research, the present study has provided a new starting point for how to measure empathy in HRI. This is crucial in order to identify which characteristics trigger empathy and, based on that, decide when to implement or avoid those characteristics.

## Data Availability

The datasets presented in this study can be found in online repositories. The names of the repository/repositories and accession number(s) can be found below: https://osf.io/sjr5p/.

## Footnotes

1
https://doi.org/10.17605/OSF.IO/4WCKY.

2 Images retrieved from: http://abotdatabase.info/collection.
